# Muscle specific declines in oxygen saturation during acute ambulation with hands-free and conventional mobility devices

**DOI:** 10.3389/fspor.2023.1210880

**Published:** 2023-12-14

**Authors:** Adam P. Bradley, Alexis S. Roehl, Joseph Smith, Ryan McGrath, Kyle J. Hackney

**Affiliations:** Department of Health, Nutrition, and Exercise Sciences, North Dakota State University, Fargo, ND, United States

**Keywords:** iWALK, axillary crutches, medical kneeling scooters, hemoglobin, muscle

## Abstract

Disuse is associated with reduced muscle oxygen saturation (SmO_2_). Improving oxygen delivery to tissues is important for healing, preventing muscle atrophy, and reducing the risk of deep vein thrombosis. Mobility devices are used during disuse periods to ambulate and protect the injured limb. This study examined SmO_2_ in walking and ambulation with various mobility devices. Thirty-eight participants randomly completed four, ten-minute trials which included: (1) walking, (2) medical kneeling scooter (MKS), (3) hands-free crutch (HFC), and (4) axillary crutch (AC). During each trial, near infrared spectroscopy sensors were placed on the vastus lateralis (VL), biceps femoris (BF), and lateral gastrocnemius (LG) of the right limb. Compared to walking, all mobility devices showed a decline in SmO_2_ in the VL of ∼10% (mean ± SD; 75% ± 12%–65% ± 17%, *P* < 0.05). In the BF, SmO_2_ declined ∼9% in AC compared to walking (76% ± 12%–67% ± 17%, *P* = 0.025). In the LG, SmO_2_ declined in AC (64% ± 16%) compared to MKS (70% ± 15%, *P* = 0.005). There were no differences in LG SmO_2_ compared to walking (69% ± 13%) in MKS (*P* > 0.05) or HFC (65% ± 15%, *P* > 0.05). In young, healthy volunteers, the use of mobility devices altered muscle oxygenation in several muscles. AC reduced muscle oxygenation in the VL, BF, and LG; while MKS and HFC maintained BF and LG muscle oxygenation at a level consistent with ambulatory walking.

## 1. Introduction

Millions of Americans annually experience lower body injury requiring a period of musculoskeletal unloading (1). These individuals face a multitude of physical consequences including muscular atrophy (2), strength loss (3), and risk of deep vein thrombosis (DVT) (4). Several different ambulation devices have been developed to assist individuals during periods of musculoskeletal unloading including traditional axillary crutches (AC) (5) and medical kneeling scooters (MKS) (6). In addition, a hands-free crutch (HFC) was recently developed as an orthopedic aid, which closely replicates the gait of normal walking while still unloading the ankle and foot (7). Previous research has investigated the effects of HFC on muscle activity. For instance, Dewar et al. showed increased levels of muscle activity in the rectus femoris (RF), gluteus maximus, and lateral gastrocnemius (LG) in HFC compared to AC (8). In an companion investigation, RF, gluteus maximus, and LG peak muscle activity were elevated in HFC compared to MKS (9).

Muscle activity, measured by surface electromyography (sEMG), is linearly related to local muscle oxygen saturation (SmO_2_) with dynamic exercise and increasing load (10, 11). Oxygen delivery and uptake to the muscle via blood flow in injured muscle and bone is particularly important for multiple basic cellular processes that are important for healing (12, 13). For example, oxygen is required for aerobic metabolism in the mitochondria of skeletal muscle to generate adenosine triphosphate (ATP) (14) and the activity of many enzymes, which are involved in healing (e.g., lack of cycloxygenase-2 activity impairs bone repair) (15). A reduction in tissue oxygen also interferes with the process of collagen synthesis, and oxygen is an important signaling molecule, which regulates the expression of several angiogenic genes (16). The ATP derived through oxidative phosphorylation is also critical for muscle protein synthesis (17).

The SmO_2_ can be measured non-invasively using near-infrared spectroscopy (NIRS), which utilizes light wavelengths absorbed by oxygenated and deoxygenated hemoglobin in arterioles, capillaries, and venules (18). NIRS is significantly correlated (*r* = 0.75) to transcutaneous O_2_ measurements (19) whereby high SmO_2_ indicates an aerobic state and low SmO_2_ indicates anaerobic metabolism (20). Recently, it was reported that there was a large (22%) decline in SmO_2_ during walking in patients with peripheral artery disease, which is indicative of impaired microcirculation (21). The risk of DVT is elevated where impaired vascular function is present, and specifically during immobilization it has been associated with NIRS-derived values including deoxygenated hemoglobin (22). For instance, it was recently shown that prolonged sitting causes decreases in SmO_2_ (23). Despite prior evidence of increased risk of DVT during immobilization as measured in NIRS-derived values, it is currently unknown how mobility devices affect lower body muscle oxygenation. The purpose of this research was to measure SmO_2_ during ambulatory walking and conduct comparisons to three orthopedic mobility devices: HFC, MKS, and AC.

## 2. Method

This study recruited healthy individuals aged 18–45 years. Participants were asked to complete three sessions on separate days: (1) informed consent/fitting, (2) ambulation device practice, and (3) muscle oxygenation testing during ambulation. In session one, following written informed consent, participants completed a Physical Activity Readiness Questionnaire, DVT screening questionnaire, and additional study-specific questionnaire (24). Exclusion criteria were pregnancy, lower limb pain, recent injury, inability to self-ambulate unassisted, body mass >124.7 kg, or height outside the range of 152.4 cm–193 cm. No individuals were excluded after screening. Participants were then fit to each device according to manufacturer specifications and settings were recorded for the practice and testing sessions. A total of 40 participants were recruited (*m*_age_ = 24.3 ± 5.1 years, *m*_BMI_ = 25.7 ± 3.6 kg/m^2^). Sample size was estimated above previous studies on similar ambulatory conditions (25, 26). Retrospective analysis determined the sample size of 38 participants was >80% power for VL, BF, and LG SmO_2_ at an alpha level of 0.05. All protocols were approved by the North Dakota State University Institutional Review Board (#IRB0003736) and each subject provided written informed consent.

The four different ambulation conditions evaluated for changes in muscle oxygenation in this investigation were: (1) walking, (2) medical kneeling scooter (MKS) (Elenker, Chino, CA), (3) hands-free crutch (HFC) (iWalkFree Inc., Long Beach, CA), and (4) axillary crutches (AC) (Personal Care Products, Larchmont, NY). Therefore, in session two, each ambulation condition (except regular walking) was practiced to assure proficiency. Proficiency was determined by the following criteria: five-minutes of safe ambulation without stopping; successful turn navigation; self-expressed comfort and ambulation confidence.

In order to test the changes in muscle oxygenation during ambulation, a randomized, within-subject, cross-over experimental design was utilized to compare lower-limb SmO_2_ effects between four different ambulation conditions (walking, HFC, MKS, AC). Participants arrived at the lab the day of testing after refraining from exercise for 24-hours and caffeine for 12-hours. A stadiometer (Seca 213; Chino, CA) and digital scale (Denver Instrument DA-150; Arvada, CO) were utilized to measure height and body mass. NIRS units (Moxy Monitor, Fortiori Design LLC, Hutchinson, MN) were placed on three muscles of the right leg, which acted as the disuse limb for mobility device comparisons. The sensors were placed on the vastus lateralis (VL), biceps femoris (BF), and lateral head LG as previously described (27). These muscle were selected given the role in the stance and swing phase as of gait cycle (28) and as well as the unique positions of these muscles with mobility devices (5, 29, 30). All participants then completed a 10-minute trial of the first ambulation condition around a 30.5 m (100 ft) rectangular walkway. Given the difficulty of determining a velocity or cadence that all mobility devices (HFC, MKS, and AC) and regular walking could be completed at, participants were instructed to complete the ambulation course at a self-selected but safe pace for each trial. A five-minute rest period occurred between each trial. The procedures were repeated until all four ambulation conditions were completed in a random order. Additional details of the research design including data quantifying oxygen consumption and hemodynamics are reported elsewhere (31, 32). Following testing, data was exported from the NIRS units and averaged per 10 min. Oxygenated and deoxygenated hemoglobin values were determined by multiplying SmO_2_ by total hemoglobin (Hb) and subtracting oxygenated hemoglobin from total Hb, respectively (27).

### 2.1. Statistical analysis

Statistical analyses were completed using SPSS version 28.0 (IBM; Armonk, NY). Descriptive characteristics were presented as mean ± SD. Tukey's method was utilized to detect and remove outliers in NIRS data. Two participants had NIRS data that were determined to be outliers and were removed from the data set (*N* = 38). Analysis of variance (ANOVA) with repeated measures were used to determine muscle oxygenation differences between ambulation conditions. An alpha level of 0.05 was used to determine significance. When statistical significance was generated, Sidak post-hoc tests were used to evaluate differences between the four ambulation conditions. Partial eta squared (pη^2^) effect size estimations also included for interpretation as pη^2^ 0.2–0.12 is considered a small effect, 0.13–0.25 is a medium effect, and >0.26 is a large effect (33). Pearson correlations were also calculated for deoxygenated hemoglobin to determine associations among upper and lower body muscular for each mobility device and walking.

## 3. Results

### 3.1. Muscle oxygenation

**Figure 1:**
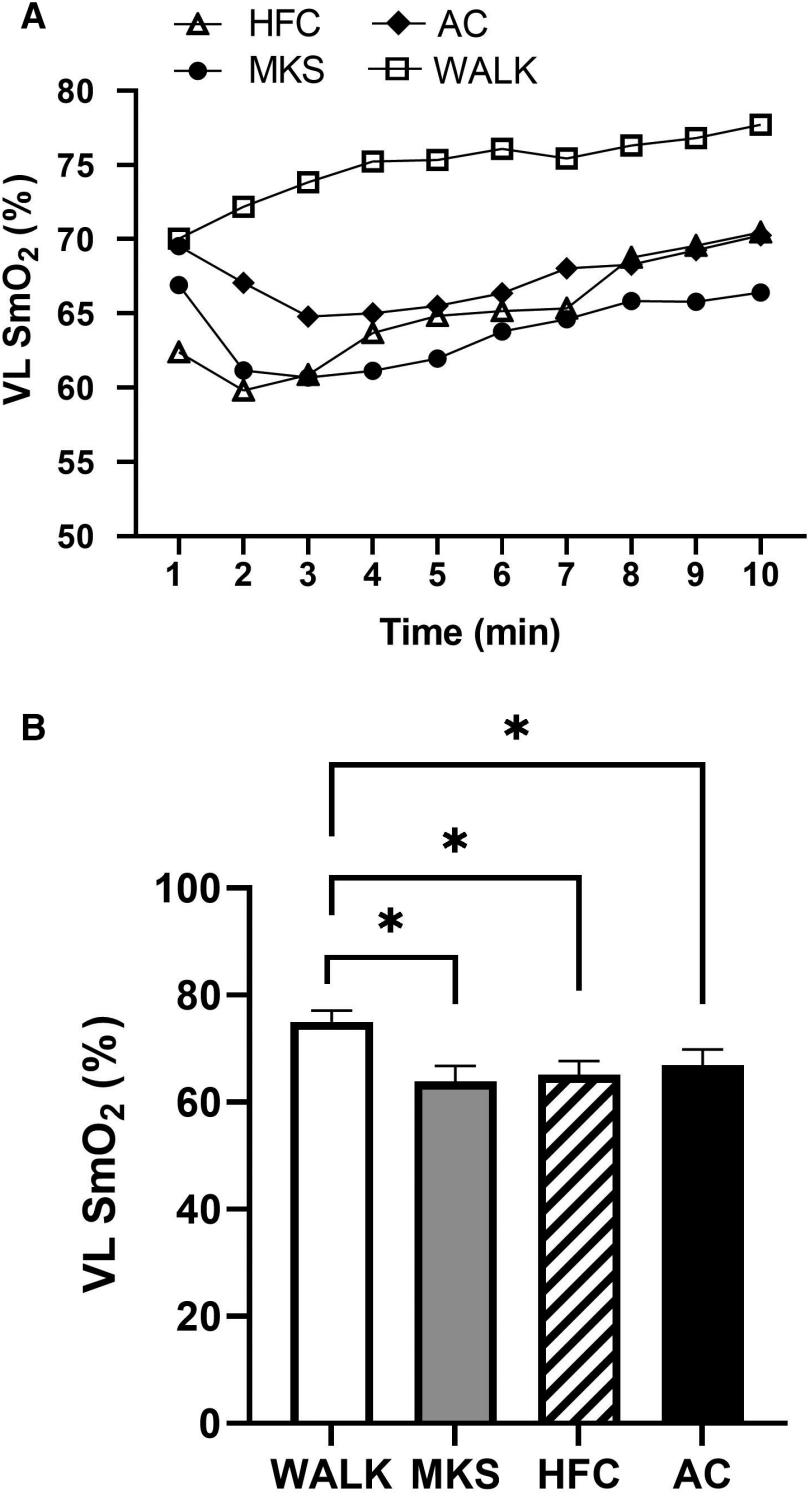
(**A**) Muscle oxygen saturation (SmO_2_) in the vastus lateralis (VL) during regular walking (WALK), medical kneeling scooter (MKS), hands-free crutch (HFC), and axial crutch (AC) over time. (**B**) Average changes in SmO_2_ for each condition. Mean ± SD. *P < 0.05.

**Figure 2:**
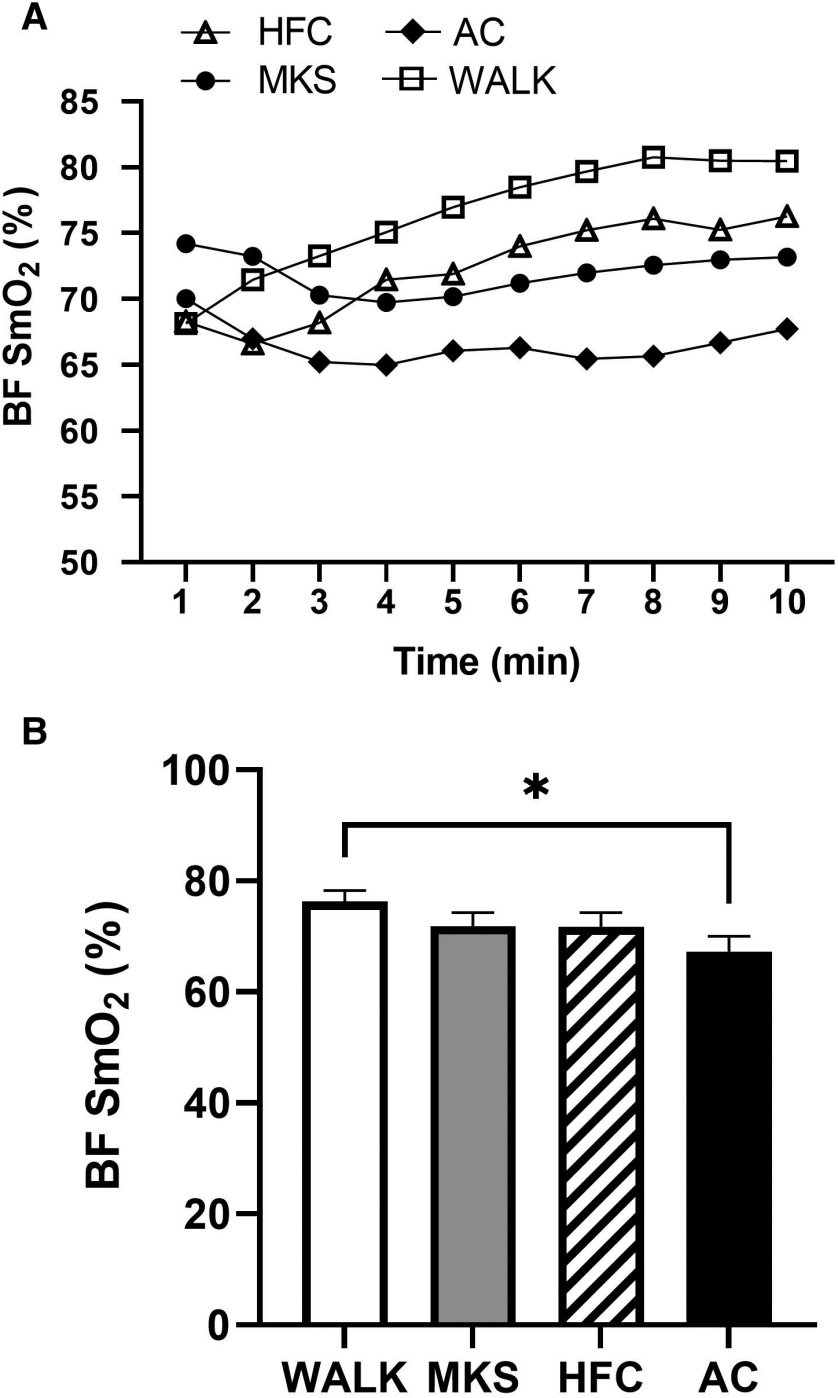
(**A**) Muscle oxygen saturation (SmO_2_) in the biceps femoris (BF) during regular walking (WALK), medical kneeling scooter (MKS), hands-free crutch (HFC), and axial crutch (AC) over time. (**B**) Average changes in SmO_2_ for each condition. Mean ± SD. *P < 0.05.

**Figure 3:**
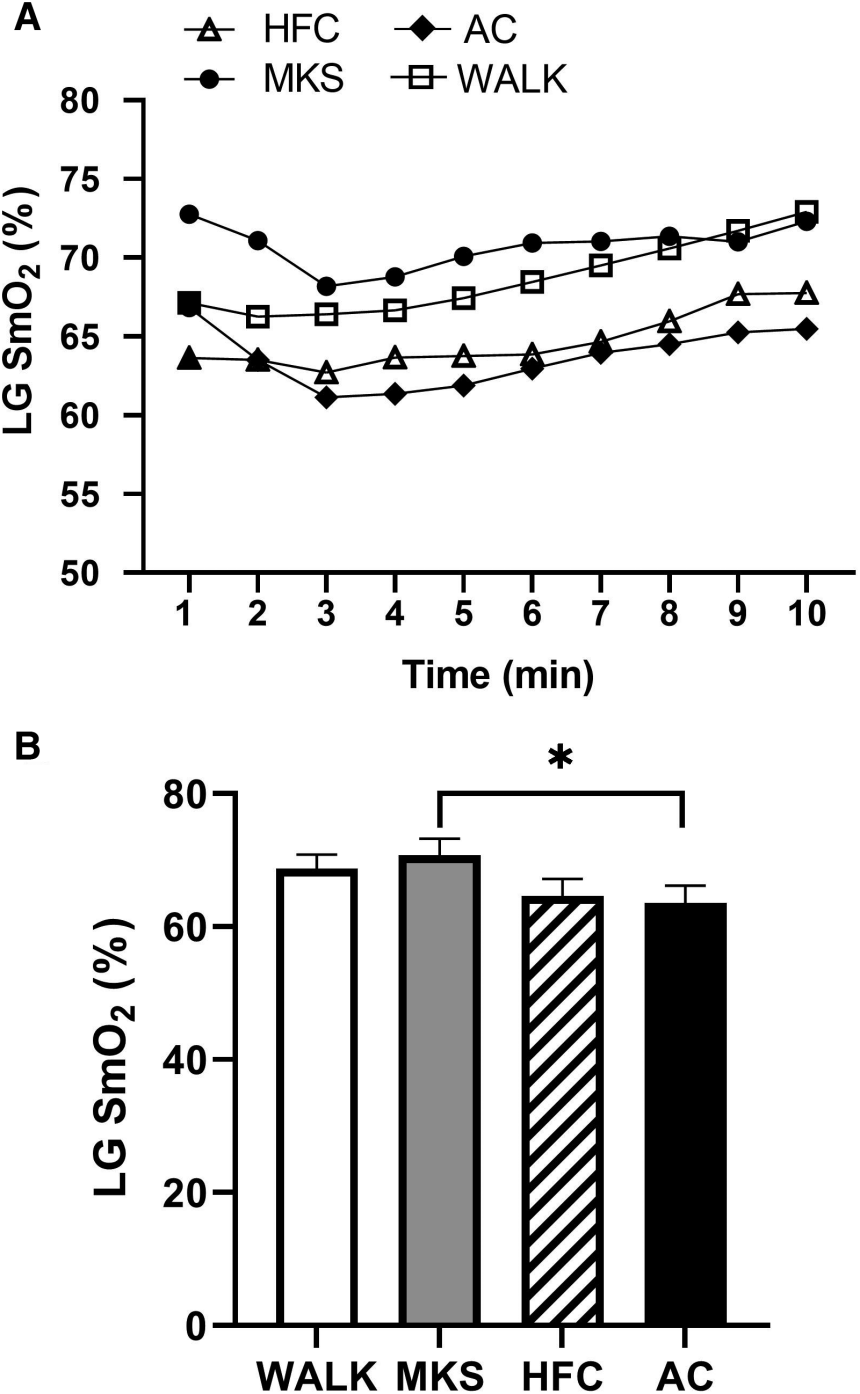
(**A**) Muscle oxygen saturation (SmO_2_) in the lateral gastrocnemius (LG) during regular walking (WALK), medical kneeling scooter (MKS), hands-free crutch (HFC), and axial crutch (AC). (**B**) Average changes in SmO_2_ for each condition. Mean ± SD. *P < 0.05.

In the VL, there were significant differences over the 10-minute trial in SmO_2_ between ambulatory and mobility device conditions (*P *< 0.001, pη^2 ^= 0.18, Figure 1). Post-hoc testing determined that when compared to walking (75% ± 13%), SmO_2_ declined in MKS (64% ± 18%, *P *= 0.002), AC (67% ± 18%, *P* = 0.047), and HFC (65% ± 16%, *P* = 0.03). In the BF, there were also significant differences over the 10-minute trial in SmO_2_ between ambulatory and mobility device conditions (*P *= 0.10, pη^2 ^= 0.10, Figure 2). Post-hoc testing determined that when compared to walking (76% ± 12%), SmO_2_ declined only in AC (67% ± 17%, *P* = 0.025). There were no significant changes in BF SmO_2_ with HFC (72% ± 15%) or MKS (72% ± 15%), *P* > 0.05). In the LG, there were significant differences in SmO_2_ between the mobility conditions (*P* = 0.011, pη^2 ^= 0.09, Figure 3). Post-hoc testing determined that SmO_2_ declined in AC (64% ± 16%) but only when compared to MKS (70% ± 15%, *P* = 0.005). There were no differences in LG SmO_2_ when compared to walking (69% ± 13%, *P* > 0.05) or when compared to HFC (65% ± 15%, *P* > 0.05).

### 3.2. Oxygenated hemoglobin

In the VL, there were significant differences over the 10-minute trial in oxygenated hemoglobin (*P* < 0.001, pη^2 ^= 0.18, Table 1). Post-hoc testing determined that when compared to walking, oxygenated hemoglobin declined in MKS (*P* = 0.002) and HFC (*P* = 0.001), but not AC (*P* = 0.051). In the BF, there were significant differences over the 10-minute trial in oxygenated hemoglobin (*P* = 0.012, pη^2 ^= 0.10, Table 1). Post-hoc testing determined that compared to walking, there was a significant decline in oxygenated hemoglobin in AC (*P* = 0.036). In the LG, there were significant differences over the 10-minute trial in oxygenated hemoglobin (*P* = 0.009, pη^2 ^= 0.10, Table 1). AC had significantly less oxygenated hemoglobin when compared to MKS (*P* = 0.002).

**Table 1:** Vastus lateralis (VL), biceps femoris (BF), and lateral gastrocnemius (LG) oxygenated (OxHb), deoxygenated (De-OxHb), and total hemoglobin (HB).

	WALK	MKS	HFC	AC
VL				
OxHb	9.12 ± 1.46	7.77 ± 2.03^a^	7.87 ± 1.83^a^	8.13 ± 2.06
De-OxHb	2.91 ± 1.49	4.25 ± 2.20^a^	4.20 ± 1.93^a^	3.97 ± 2.15^a^
Total Hb	12.02 ± 0.41	12.02 ± 0.52	12.07 ± 0.50	12.15 ± 0.43^a^
BF				
OxHb	9.14 ± 1.48	8.62 ± 1.75	8.65 ± 1.77	8.10 ± 1.94^a^
De-OxHb	2.83 ± 1.45	3.40 ± 1.84	3.41 ± 1.88	3.96 ± 2.05^a^
Total Hb	11.90 ± 0.42	12.00 ± 0.42	12.01 ± 0.41	12.04 ± 0.34^a^
LG				
OxHb	8.33 ± 1.51	8.67 ± 1.59	7.96 ± 1.57	7.74 ± 1.82^b^
De-OxHb	3.71 ± 1.66	3.49 ± 1.84	4.22 ± 1.83	4.47 ± 2.04^b^
Total Hb	12.03 ± 0.50	12.17 ± 0.48^a^	12.18 ± 0.49^a^	12.21 ± 0.46^a^

*N* = 38.

^a^ Significantly different vs. WALK.

^b^ Significantly different MKS; Mean ± SD; *P* < 0.05.

### 3.3. Deoxygenated hemoglobin

In the VL, there were significant differences over the 10-minute trial in deoxygenated hemoglobin (*P* < 0.001, pη^2 ^= 0.17, Table 1). Post-hoc testing determined that when compared to walking, deoxygenated hemoglobin increased in MKS (*P* = 0.001), HFC (*P* = 0.03), and AC (*P* = 0.003). In the BF, there were significant differences over the 10-minute trial in deoxygenated hemoglobin (*P* = 0.005, pη^2 ^= 0.11, Table 1). Post-hoc testing determined that compared to walking, there was a significant increase in deoxygenated hemoglobin in AC (*P* = 0.015). In the LG, there were significant differences over the 10-minute trial in deoxygenated hemoglobin (*P* = 0.005, pη^2 ^= 0.11, Table 1). AC had significantly greater deoxygenated hemoglobin when compared to MKS (*P* = 0.002).

During walking, VL deoxygenated hemoglobin was significantly correlated with BF (*r* = 0.456, *P* = .004) and LG (*r* = .515, *P* < 0.001) deoxygenated hemoglobin. BF deoxygenated hemoglobin was also significantly correlated with LG deoxygenated hemoglobin (*r* = 0.602, *P* < 0.001). During HFC, VL deoxygenated hemoglobin was significantly correlated with BF (*r* = 0.619, *P* < 0.001) and LG (*r* = 0.513, *P* < 0.001) deoxygenated hemoglobin. BF deoxygenated hemoglobin was also significantly correlated with LG deoxygenated hemoglobin (*r* = 0.392, *P* = 0.015). During MKS, VL deoxygenated hemoglobin was significantly correlated with BF (*r* = 0.494, *P* = 0.002) and LG (*r* = 0.532, *P* < 0.001) deoxygenated hemoglobin. BF deoxygenated hemoglobin was also significantly correlated with LG deoxygenated hemoglobin (*r* = 0.396, *P* = 0.015). During AC, VL deoxygenated hemoglobin was significantly correlated with BF (*r* = 0.563, *P* < 0.001) and LG (*r* = 0.719, *P* < 0.001) deoxygenated hemoglobin. BF deoxygenated hemoglobin was also significantly correlated with LG deoxygenated hemoglobin (*r* = 0.606, *P* < 0.001).

### 3.4. Total hemoglobin

In the VL, there were significant differences over the 10-minute trial in total hemoglobin (*P* < 0.045, pη^2 ^= 0.70, Table 1). Post-hoc testing determined that when compared to walking, total hemoglobin increased in AC (*P* = 0.016). In the BF, there were significant differences over the 10-minute trial in total hemoglobin (*P* < 0.001, pη^2 ^= 0.15, Table 1). Post-hoc testing determined that compared to walking, there was a significant increase in total hemoglobin in AC (*P* < 0.001). In the LG, there were significant differences over the 10-minute trial in total hemoglobin (*P* = <0.001 pη^2 ^= 0.31, Table 1). Post-hoc testing determined that compared to walking, there were increases in total hemoglobin in AC (*P* < 0.001), HFC (*P* < 0.001), and MKS (*P* < 0.001).

## 4. Discussion

The purpose of this study was to measure lower extremity SmO_2_ in walking compared to ambulation with common mobility devices. The primary findings of this acute disuse simulation were that we observed muscle and mobility device-related: (1) declines in muscle oxygenation, (2) elevations in de-oxygenated hemoglobin, and (3) increases in total hemoglobin. These acute alterations were evident in as little as 10 min of mobility device use when compared to walking. The results of this study may have implications for increased healing, reduced muscle loss, and mitigation of the DVT.

Oxygen delivery plays a crucial role in wound and fracture healing by generating ATP via aerobic metabolism and stimulating the activity of critical enzymes for repair (12, 13). The current investigated reports, when compared to normal walking, all mobility devices tested showed an average decline in SmO_2_ in the VL of ∼−10%. We also report that in AC only ambulation there were declines in SmO_2_ in the BF (−9%) when compared to walking and LG (−6%) when compared to MKS. There are two physiological mechanisms that could explain why there was an acute decline in muscle oxygenation when using mobility devices. First, an inverse linear relationship has previously been demonstrated between dynamic muscle activation and SmO_2_ with demanding exercise (10, 11). Greater dynamic muscle activation and aerobic energy metabolism can subsequently decrease in SmO_2_, as local oxygen delivery and uptake is below the capabilities of aerobic metabolism in the muscle (34). A secondary mechanism for reduced SmO_2_ when using the mobility devices is that with muscle inactivity SmO_2_ can also decline below resting levels given reduced blood flow and lack of oxygen delivery to the muscle (23). Therefore, the purported declines in SmO_2_ in current investigation with mobility device use may be explained either by very high local muscle energy requirements or reduced blood flow and oxygen delivery.

In our investigation, we cannot definitely distinguish which mechanism is driving the reported acute declines in muscle oxygenation. In a companion paper, we have shown the total body oxygen consumption (VO_2_) was higher (∼35%) when using the mobility devices when compared to walking (31). However, total body VO_2_ represents the summation of aerobic energy metabolism of all contracting skeletal muscles and other physiological systems during movement (35). Therefore, contributions from additional upper extremity dynamic muscle activation during AC, increased activity of dynamic bilateral leg muscles during MKS propulsion, or dynamic hip flexor activation in lifting the HFC can drive increases in total VO_2_. Further, if even considering the additional dynamic muscle activity and aerobic energy metabolism of other physiological systems in the previous study, VO_2_ values ranged from 15 to 20 ml/kg/min using the mobility devices compared to 13 ml/kg/min with regular walking (31). Crum et al. showed that aerobic power with dynamic exercise needed to reach a VO_2_ threshold of 36 ml/kg/min before SmO_2_ started to decline given dynamic muscle activation and accelerated aerobic energy metabolism (34). In addition, Vasquez-Bonilla et al. suggest SmO_2_ values may need to decline below 26% before anaerobic metabolism becomes the predominant energy system (36). Thus, we speculate the local muscle oxygenation saturation declines with mobility device use in the present study were a result of reduced local blood flow and oxygen delivery during the 10-minute disuse simulation given the greatest decline in SmO_2_ with the mobility devices use was ∼10%.

The VL in particular showed a mean decline of 10% when using all mobility devices when compared to walking. Given the VL is primarily a knee extensor, the isometric/stabilization demands placed on VL when ambulating with a fixed knee angle like in HFC and MKS differ from the dynamic demands of unassisted walking. Higher isometric knee extensor activation has been demonstrated at longer muscle lengths, such as the 90° knee angle in HFC and MKS (37). Although muscle activity is higher during isometric activation, ATP splitting and subsequence oxygen utilization to resynthesize ATP is lower in isometric muscle actions compared to dynamic activation given reduced cross-bridge cycling in the muscle (e.g., Fenn Effect) (38). Further, when muscles are stretched such as with VL during mobility device use, ATP splitting is further reduced even lower than with isometric muscle actions (39). A previous investigation into muscle activation with walking and HFC have shown similar patterns of muscle activation in the VL (40). However, these muscle activation pattern similarities may not have been influential enough to elicit similar SmO_2_ responses to walking in this investigation with any of the mobility devices tested. Declines in SmO_2_ in BF and LG when ambulating with AC only also suggest a trend towards an anaerobic local muscle environment given total musculoskeletal unloading from ground reaction forces in the suspended limb. Given that BF and LG function as knee flexors, lower limb SmO_2_ decreases may also be explained via isometric knee flexion demands (40). We speculate that SmO_2_ declined because none of the muscles evaluated in the present study (VL, BF, LG) were contracting through a full range of motion as with regular walking. This reduces ATP splitting in muscle cross-bridge cycling and subsequent adenosine diphosphate build up which triggers additional oxygen uptake into the muscle (38).

Indicators of deoxygenated hemoglobin is associated with increased risk of DVT (22). In brief, increases in deoxygenated hemoglobin were the reverse of the declines in SmO_2_. For example, in the VL, deoxygenated hemoglobin increased in MKS, HFC, and AC when compared to walking. In the BF, there was a significant increase in deoxygenated hemoglobin in AC only vs. walking and in the LG, AC had significantly greater deoxygenated hemoglobin when compared to MKS only. Correlations between upper leg musculature (VL, BF) and lower extremity muscular (LG) showed positive and significant associations in walking, HFC, MKS, and AC; however, the largest correlation occurred in AC between the VL and LG (*r* = .719). One attractive hypothesis for the differences observed is “functional sympatholysis”, whereby, metabolites from local contracting skeletal muscle interrupts pathways mediating sympathetic vasoconstriction (41). In a previous experiment, mild to moderate exercise (handgrip 20%–33% of maximum effort) maintained muscle oxygenation in exercising muscles, but led to decreases in oxygenation in resting muscle (42). Thus, given lower extremity muscles (VL, BF, LG) are active through a larger range of motion during walking, local metabolites may have maintained vasodilation and lower levels of deoxygenated hemoglobin. In contrast, the activity of these muscle may be less or even at rest with mobility device use, thus, the lack of local metabolite build-up in arterioles could lead to sympathetic vasoconstriction; which increased deoxygenated hemoglobin.

Total hemoglobin has been used as a non-invasive indicator of muscle blood volume changes (43, 44) and there were subtle changes in total hemoglobin in the disuse simulation. Total hemoglobin elevations appeared to be the most persistent with AC as increases were observed in VL and BF when compared to walking. In the LG, the muscle where volume changes would be the most expected with mobility device use, there were increases in total hemoglobin compared to walking in the following order: MKS (+1.16%), HFC (+1.25%), and AC (+1.50%). These data suggest a low but significant level of venous blood pooling in the lower extremity muscles that were unloaded in 10 min of ambulation.

Prolonged AC use has shown elevated calf circumferences (45), which is another indicator of blood pooling in the lower extremity. The consequence of blood pooling during immobilization of more than 48 h is strongly associated with DVT risk (45). Muscle pump activity is also a critical factor for preventing stasis (25) as knee positions at 90 degrees flexion are associated with reduced flow rates (26). However, in a previous investigation, we reported post-mobility device ambulation popliteal venous blood flow rates via diagnostic ultrasound (32). All mobility devices had some level of reduced venous flow; however, only significant changes were detected in the MKS vs. traditional walking (32). Given that NIRS data were collected during mobility device ambulation and blood flow indicators were taken immediately after ambulation, these data cannot be directly correlated. Further investigation of blood volume and stasis are warranted with mobility device use in both acute and chronic conditions.

## 5. Conclusion

Local muscle oxygenation to the muscles of the lower extremity is reduced acutely with mobility device ambulation, and the response is specific to the muscle and mobility device. All mobility devices showed reduced muscle oxygenation in the VL. Muscle oxygen saturation levels in the BF and LG muscles while using the MKS and HFC devices were similar to those of ambulatory walking. Reduced muscle oxygenation, increased de-oxygenation, and elevated total hemoglobin during use of AC were recorded in all three muscles tested. A strength of the investigation was the use of NIRS during acute mobility device use (19), including novel HFC applications. This is first investigation to include NIRS in evaluation of mobility devices use and provides muscle oxygenation patterns that may be more beneficial for tissue repair compared to surface EMG. A limitation of the investigation was that the disuse simulation was performed in healthy adults in the absence of injury or post-operative stressors. In support of our acute findings, Rambani et al. has provided one of the only randomized controlled trials in this area and reported average hospital stay duration in patients using HFC were shorter (∼7 days) with greater musculoskeletal functional assessments compared to crutches (7). Future large-scale clinical trials in patients with injury are needed to further illicit if the acute changes in muscle oxygenation may be altered given inflammatory mediated disruptions in blood flow.

## Data Availability

The datasets presented in this study can be found in online repositories. The names of the repository/repositories and accession number(s) can be found below: https://docs.google.com/spreadsheets/d/1hBFlrnhM9BRvsYe7N4IZi0zfkosG6cCP/edit?0usp=share_link&ouid=112900476014567350397&rtpof=true&sd=true.
